# Supplementary material for: Incidence of Hospitalisation and Emergency Department Visits for Pneumococcal Disease in Children, Adolescents, and Adults in Liguria, Italy: A Retrospective Analysis from 2012–2018
Source: Vaccines (Basel). 2022 Aug 24;10(9):1375. doi: 10.3390/vaccines10091375 (PMC9504569; doi:10.3390/vaccines10091375)
Supplement: Supplementary file 1 [file vaccines-10-01375-s001.zip › vaccines-1802293-SI.pdf]

Incidence of Hospitalisation and Emergency Department Visits for Pneumococcal Disease in Children, Adolescents, and Adults in Liguria, Italy: A Retrospective Analysis From 2012–2018

Matteo Astengo[1,*], Chiara Paganino[1], Daniela Amicizia[1,2], Laura Sticchi[2,3], Andrea Orsi[2,3], Giancarlo Icardi[2,3], Maria Francesca Piazza[1], Salini Mohanty[4], Francesca Senese[5], Gian Marco Prandi[5], Filippo Ansaldi[1,2]

[1] Regional Health Agency of Liguria (ALiSa), Genoa, Italy

[2] Department of Health's Science (DiSSal), University of Genoa, Genoa, Italy

[3] Hygiene Unit, San Martino Policlinico Hospital-IRCCS for Oncology and Neurosciences, 16132 Genoa, Italy.

[4]Merck & Co., Inc., Rahway, New Jersey, USA; [5]MSD Italy, Via Vitorchiano 151, 00189 Rome, Italy.

***Correspondence:**

Matteo Astengo

Regional Health Agency of Liguria (ALiSa), Genoa, Italy

Piazza della Vittoria 15, 16121 Genoa Italy.

Tel: +39 0105488257

E-mail: Matteo.astengo@regione.liguria.it

Table S1. Search terms and diagnoses of IPD and pneumonia used to identify episodes all patients in Liguria, Italy (2012–2018)

Diagnosis	Description	ICD-9-CM codes
Pneumonia (specified or all-cause)	Pneumococcal pneumonia	481
	Pneumococcal infection and bacterial pneumonia, bronchopneumonia or organism unspecified pneumonia	041.2 and 482.9, 485, or 486
Pneumonia (all-cause)	Viral pneumonia	480.0-480.3, 480.8, or 480.9
	Pneumonia due to other specified organism	483.0, 483.1, or 483.8
	Pneumonia in infectious diseases classified elsewhere	484.1, 484.3, or 484.5-484.8
	Influenza with pneumonia	487.0
Pneumonia (unspecified or all-cause)	Unspecified bacterial pneumonia, bronchopneumonia or pneumonia	482.9, 485, or 486
IPD	Pneumococcal septicaemia	038.2
	Pneumococcal infection and bacteraemia or septicaemia	041.2 and 790.7, 038.9, or 038.0
	Pneumococcal meningitis	320.1
	Pneumococcal infection and meningitis	041.2 and 320.2, 320.82, 320.9, or 322.9
	Pneumococcal infection and empyema or pleural effusion	041.2 and 510.9, 511.1, or 511.9
	Bacteraemic pneumonia: pneumococcal-specific	038.2<br>038.0 and 041.2<br>038.9 and 041.2<br>790.7 and 041.2
	Bacteraemic pneumonia: pneumococcal-unspecific	038.0<br>038.9<br>790.7
AOM		382.x

AOM: acute otitis media; ICD-9-CM: International Classification of Diseases, Ninth Revision, Clinical Modification; IPD: invasive pneumococcal disease.

Figure S1. Age-stratified annual incidence of (A) unspecified pneumonia and (B) pneumococcal pneumonia in adolescents and adults ≥15 years of age following the introduction of PCV13 in Liguria, Italy (2012–2018)

A.

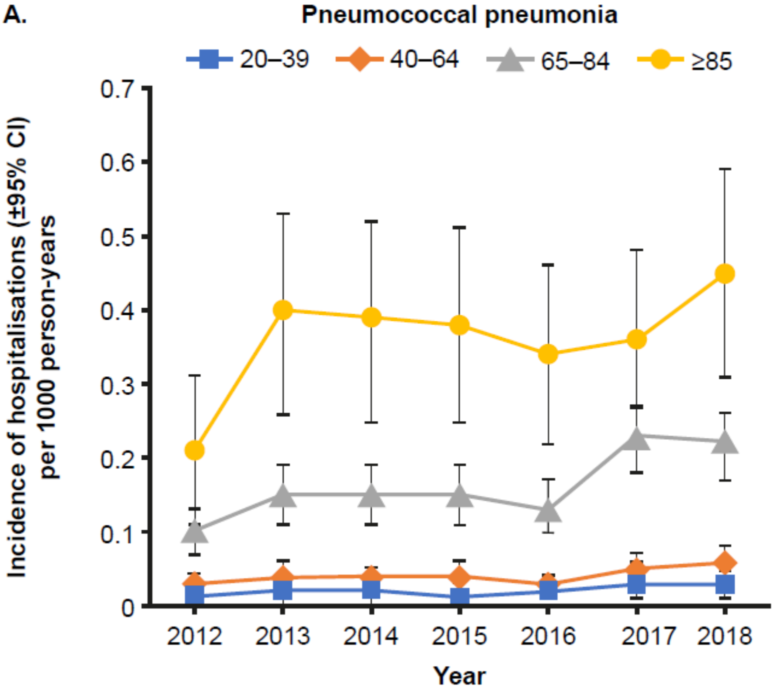

B.

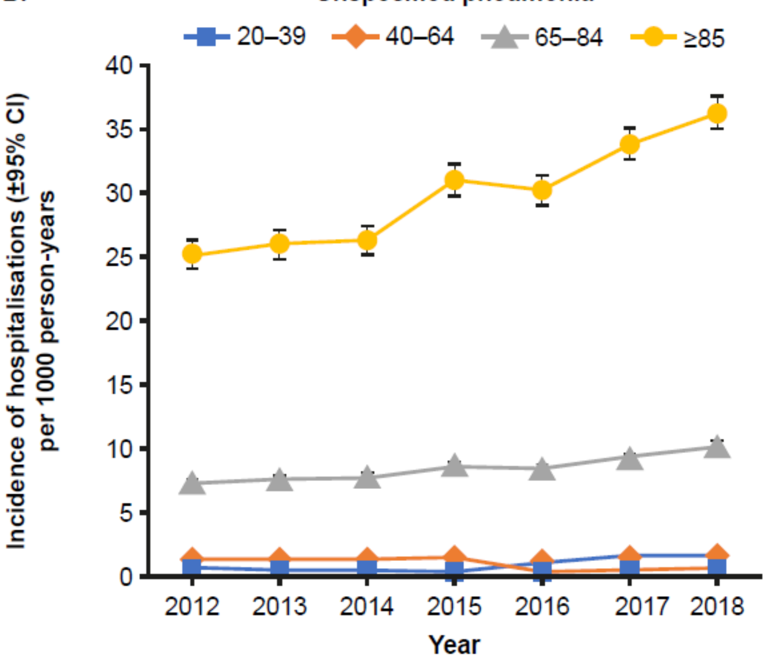

CI: confidence interval; PCV13: 13-valent pneumococcal conjugate vaccine.

Figure S2. Age-stratified annual incidence of IPD in adolescents and adults ≥15 years of age following the introduction of PCV13 in Liguria, Italy (2012–2018)

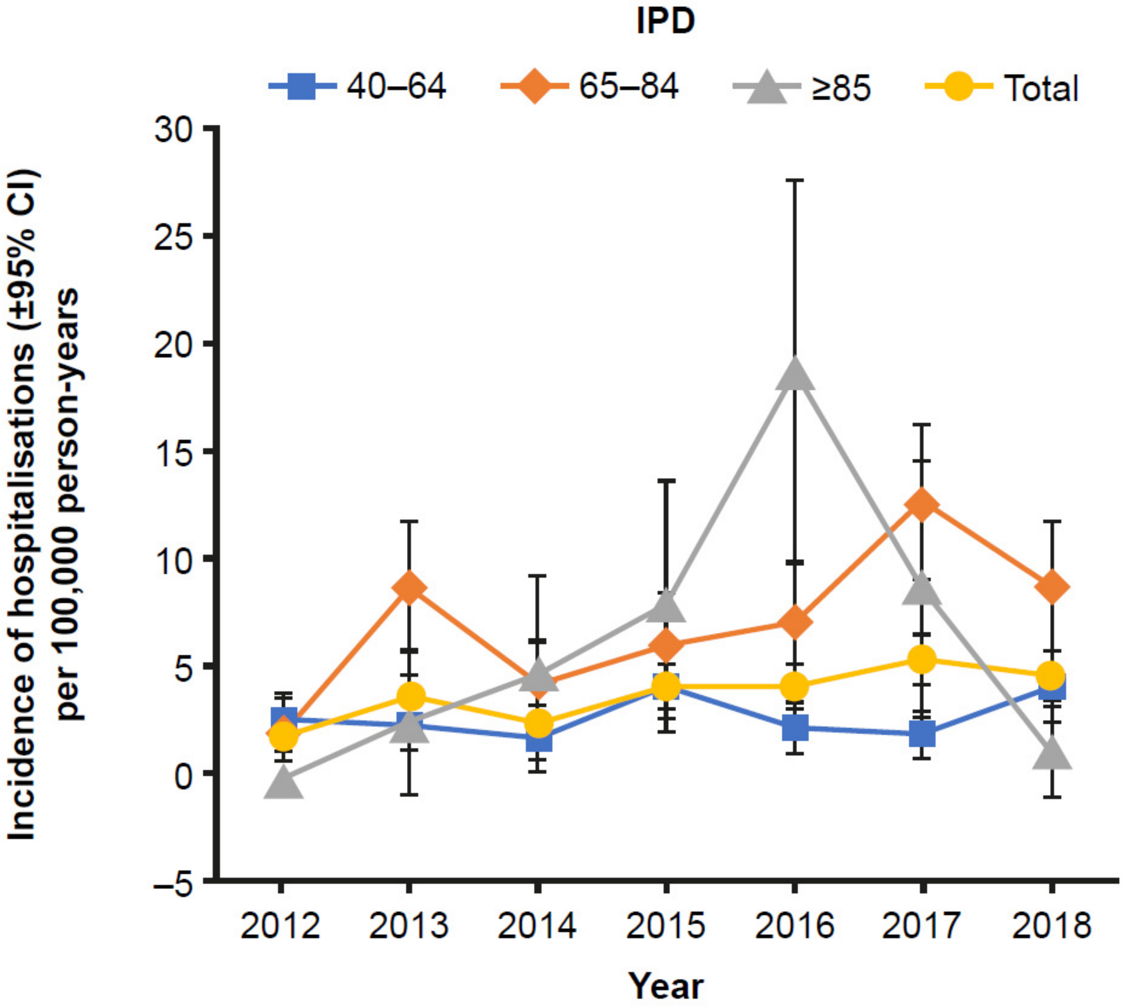

CI: confidence interval; IPD: invasive pneumococcal disease; PCV13: 13-valent pneumococcal conjugate vaccine.
